# Persistence of environmental DNA in cultivated soils: implication of this memory effect for reconstructing the dynamics of land use and cover changes

**DOI:** 10.1038/s41598-020-67452-1

**Published:** 2020-06-29

**Authors:** Anthony Foucher, Olivier Evrard, G. Francesco Ficetola, Ludovic Gielly, Julie Poulain, Charline Giguet-Covex, J. Patrick Laceby, Sébastien Salvador-Blanes, Olivier Cerdan, Jérôme Poulenard

**Affiliations:** 1 Laboratoire Des Sciences du Climat Et de L’Environnement (LSCE/IPSL), UMR 8212 (CEA/CNRS/UVSQ), Université Paris-Saclay - Orme Des Merisiers, 91191 Gif-sur-Yvette Cedex, France; 2 Department of Environmental Science and Policy, Università Degli Studi Di Milano, Milan, Italy; 3 Laboratoire d’Écologie Alpine, CNRS, Université Grenoble Alpes, Grenoble, France; 4 Génomique Métabolique, Genoscope, Institut François Jacob, CEA, CNRS, Univ Evry, Université Paris-Saclay, Evry, France; 5 Université Grenoble Alpes, Université Savoie Mont Blanc, CNRS, EDYTEM (Environnements, DYnamiques Et TErritoires de La Montagne), Chambéry, France; 6 Environmental Monitoring and Science Division (EMSD), Alberta Environment and Parks (AEP), Calgary, AB Canada; 7 Laboratoire GéoHydrosystèmes Continentaux (GéHCO), E.A 6293, Faculté des Sciences et Techniques, Université F. Rabelais de Tours, Parc de Grandmont, 37200 Tours, France; 8 Département Risques et Prévention, Bureau de Recherches Géologiques et Minières (BRGM), 3 avenue Claude Guillemin, 45060 Orléans, France

**Keywords:** Biodiversity, Agricultural genetics, Plant genetics, Ecosystem ecology

## Abstract

eDNA refers to DNA extracted from an environmental sample with the goal of identifying the occurrence of past or current biological communities in aquatic and terrestrial environments. However, there is currently a lack of knowledge regarding the soil memory effect and its potential impact on lake sediment eDNA records. To investigate this issue, two contrasted sites located in cultivated environments in France were studied. In the first site, soil samples were collected (n = 30) in plots for which the crop rotation history was documented since 1975. In the second site, samples were collected (n = 40) to compare the abundance of currently observed taxa versus detected taxa in cropland and other land uses. The results showed that the last cultivated crop was detected in 100% of the samples as the most abundant. In addition, weeds were the most abundant taxa identified in both sites. Overall, these results illustrate the potential of eDNA analyses for identifying the recent (< 10 years) land cover history of soils and outline the detection of different taxa in cultivated plots. The capacity of detection of plant species grown on soils delivering sediments to lacustrine systems is promising to improve our understanding of sediment transfer processes over short timescales.

## Introduction

Environmental DNA (eDNA) is a complex mixture of genetic material present in environmental samples.^1–4^ During the last several decades, eDNA studies have significantly improved our ability to detect a range of organisms, including macroorganisms, plants, animals and even to reconstruct paleo communities.^5,6^ Detection of taxa present in an eDNA sample can be accessed with a general approach based on target PCR (polymerase chain reaction) called “DNA metabarcoding”.^7^ This method was successfully applied in a wide range of environmental samples collected from soil compartments,^8^ freshwater ecosystems,^9,10^ marine environnements^11^ as well as for palaeoenvironnemental reconstructions of aquatic and terrestrial ecosystems.^12–14^

eDNA studies conducted on soils are assumed to analyze particles of eDNA originating from organism remains, organic matter, or from extra-cellular DNA molecules bound to soil compounds, such as clay.^15^ In principle, eDNA can remain in soil or sediments for long periods as eDNA bound to clay was shown to be protected against degradation.^4^ eDNA has been successfully extracted from lake^13,16–18^ and cave sediment to identify the organisms living in the past.^19^ The potential of this method has also been demonstrated for sediment source fingerprinting.^20^ However, to our knowledge, limited information is available on the DNA memory effect in soils in general, and in intensively cultivated areas in particular.^8,21^

The long-term persistence of plant DNA was investigated in Alpine soils with DNA metabarcoding by Yoccoz et al.^8^ In this study, soil core samples were collected in formerly cultivated plots that were abandoned between 1810 and 1986 in temperate environments. These authors found that the number of crop DNA sequences retrieved strongly varied with years since the last cultivation period. They observed a negative relationship between the number of DNA sequences and the number of years after crop abandonment with the detection of DNA sequences disappearing completely after approximately 50 years. Furthermore, Yoccoz et al. ^8^ demonstrated the absence of DNA sequences in a meadow that was never cultivated located 1 km away from the formerly cultivated plots. This result suggests that the locally produced biomass contributes the large majority of the soil DNA at a given location and that long-distance transport can be not significant^22^.

Aside from these limited studies into the DNA memory effect in soils, according to Taberlet et al.,^4^ the general lack of knowledge for eDNA in soils outside of the microbial world may be due to the fate and behavior of DNA in the terrestrial soil matrix. In particular, the processes determining long-term persistence of eDNA remain poorly understood. The detectability and persistence of eDNA in soils can be influenced by many factors such as the DNA molecule structure or environmental conditions (e.g. temperature, pH, UV radiation, and microbial or enzymatic activities) (see references in Taberlet et al.^4^) with more rapid degradation in tropical environments. Indeed, there are many fundamental research questions that require further investigation. How long can DNA persist in surface soils? How does it impact our capacity to characterize plant communities in cultivated areas? What is the impact of agricultural practices (e.g. soil tillage) on DNA preservation? These questions are of primary importance to improve our understanding of what DNA is potentially recorded in soils and in sediments collected in riverine and lacustrine systems, along with what may be revealed by palaeoenvironmental reconstructions based on sedimentary sequences or sediment source fingerprinting studies.

**Figure 1:**
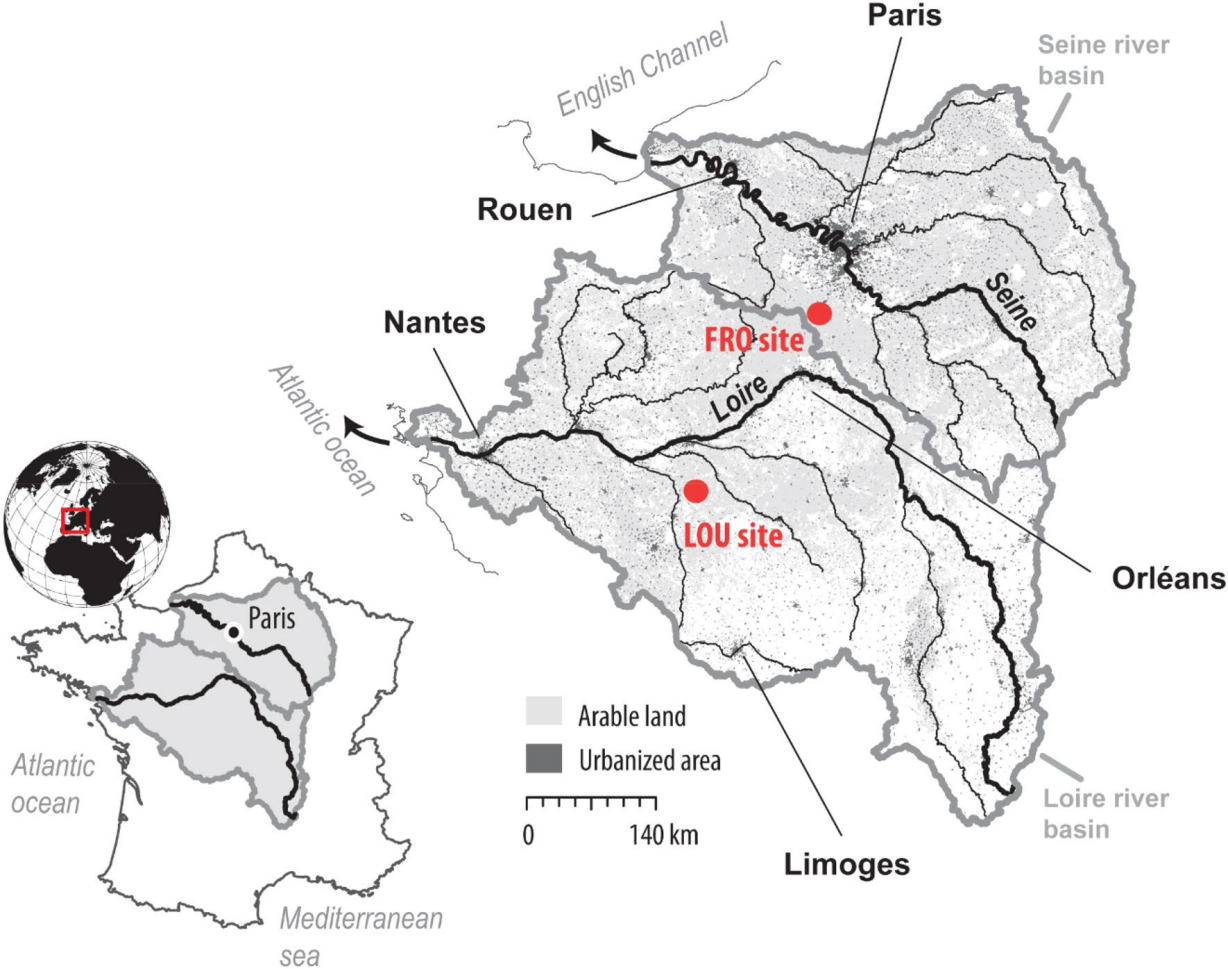
Localization of the studied sites of Fromonvilliers (FRO site) and Le Louroux (LOU site) within the Seine and the Loire River basins. (The map was created using the free software QGIS 3.12.2 (https://www.qgis.org). Land use information were provided by Corine land cover data^44^ and hydrological information by the BD Cartage database https://www.sandre.eaufrance.fr).

The capacity of soils to retain amplifiable DNA molecules over long temporal periods remains a fundamental question. Accordingly, the objective of this study is to investigate the memory effect of eDNA in the terrestrial soil matrix (i.e. the capacity of soil to archive the information of past cultivated plants) through the analysis of soil samples collected in two agricultural catchments located in the most intensively cultivated region of Central France (Fig. 1). At the first site (Fromonvilliers site), soil samples were collected in plots where the crop rotation history was well documented since 1975 to provide insights into the persistence of eDNA. In addition, these samples were collected in fields where either conventional (e.g. soil tillage) or alternative practices (i.e. no tillage and direct seeding) were implemented in order to investigate the impact of these practices on eDNA preservation. At the second site (Louroux site), the potential of eDNA for recording the current and recent land uses was tested through the comparison of the species observed in the field and those taxa detected with eDNA sequences. Finally, the impact of these results for the use of this technique to reconstruct past land use and land cover changes and to trace sediment source contributions will be discussed. Results will improve our knowledge on the applicability and the potential of eDNA metabarcoding in a wide range of environmental research questions.

## Results

### eDNA memory effect in soils

DNA analyses highlight the presence of the last cultivated crops (< 7 years) in conventional and conservation farming. They also underline the ability of these methods to detect a large variety of weeds under both agricultural practices. Results from the analyses highlighting the memory effects for both farming practices are detailed below.

#### Conventional farming

**Figure 2:**
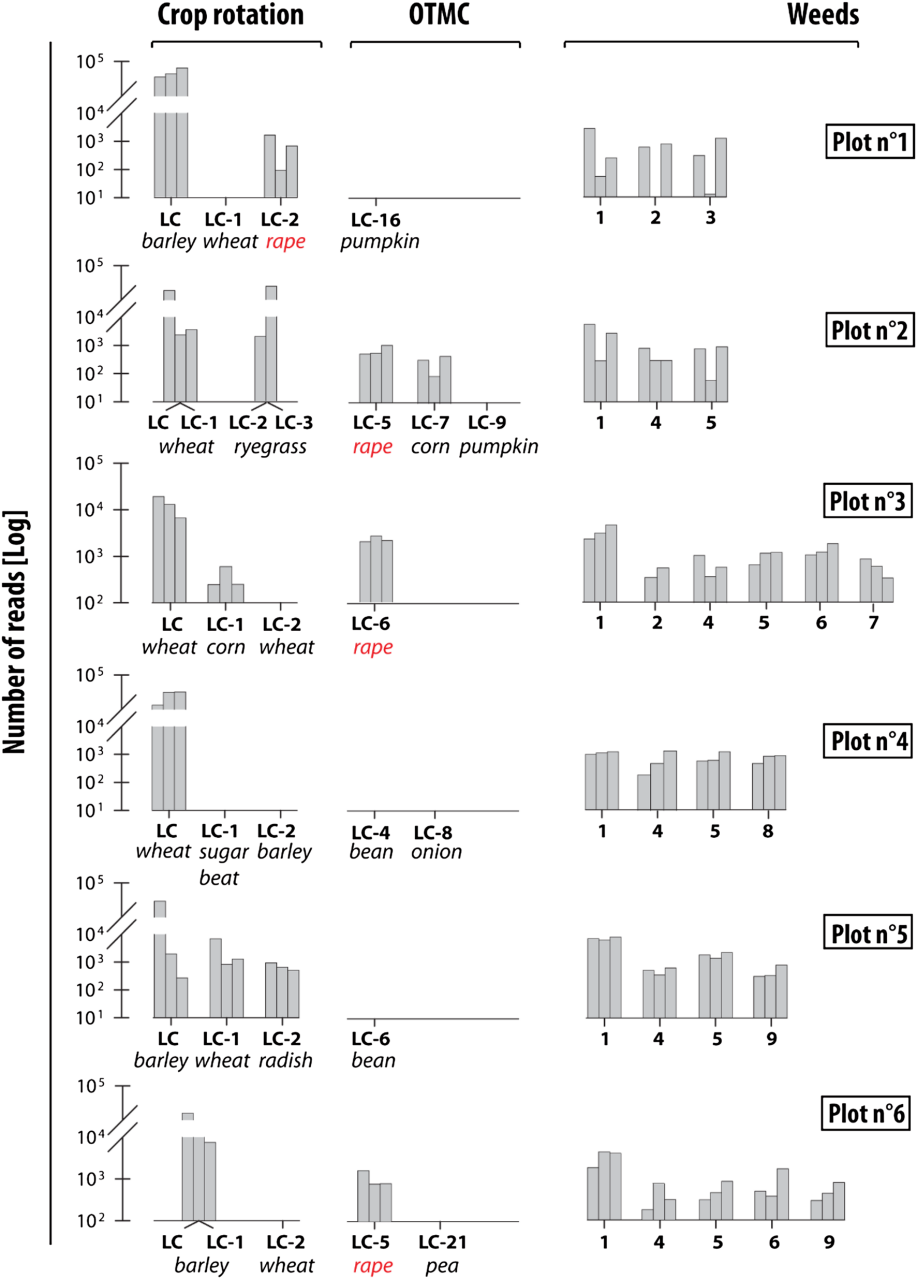
Abundance of crops and weeds taxa identified by eDNA analyses under conventional farming. The 3 bars correspond to the 3 replicates for each site. LC corresponds to the last crop and LC-X to the crop cultivated x year(s) before the last crop. OTMC corresponds to Time Marker Crops. (1) bindweed (*Convolvulus*) (2) ryegrass (*Lolium perenne*) (3) black nightshade (*Solanum nigrum*) (4) knotweed (*Fallopia convolvulus*) (5) healtyfoin (*Onobrychis viciifolia*) (6) clover (*Trifolium*) (7) buttercup (*Rananculus*) (8) oatmeal (*Avenula*) (9) field gromwell (*Lithospermum arvense*).

eDNA analyses for plot 1 have indicated the dominance of the last crop barley (*Hordeum*, MTR (Mean Total Reads) 95%, SD (Standard Deviation) 18%). Wheat (*Triticum*) cultivated in LC-1 (Last cultivated Crop: LC*-*X correspond to the crop cultivated X years before the sampling survey) was not detected whereas the LC-2 crop, rape, was identified (MTR, 1.3%, SD 0.75%). Between year LC and LC-1, soil was ploughed when direct seeding was performed between LC-2 and LC-1. The OTMC (One Time Marker Crop) identified for this plot in LC-16 (pumpkin, *Cucurbita maxima*) was not detected. In plot 1, eDNA analyses detected only two crop species (barley and rape) and three weed species (bindweed (*Convolvulus*), ryegrass (*Lolium perenne*) and black nightshade (*Solanum nigrum*)) (Fig. 2).

For plot 2, wheat was cultivated during two successive years (LC and LC-1). The field was then occupied by an artificial grassland for 2 years (i.e. LC-2 and LC-3). During this period, this plot was converted into grassland and covered with a weed, ryegrass. eDNA was strongly variable among the three samples collected in this field with the first replicate demonstrating the dominance (75%) of the last crop whereas sample three indicated the dominance of grassland weeds (91%) (Fig. 2). For this plot, three OTMC were known. The OTMC in year LC-5 (rape) was clearly identified (MTR 2.5%, SD 1.7%) along with the OTMC LC-7 (corn (*Zea mays*)—MTR 1%, SD 0.75%). Contrarily, the LC-9 OTMC (pumpkin) was not identified. In addition to ryegrass, the weed population included three other taxa in plot 2 including bindweed, knotweed (*Fallopia convolvulus*), healtyfoin (*Onobrychis viciifolia*).

Results for plot 3 indicate the dominance of the last crop, wheat (MTR 42%, SD 10%) (Fig. 2). The LC-1 crop (corn) was detected in lower proportions (MTR 1.1%, SD 1.7%). Between the LC and LC-1, the soil was ploughed. The DNA results demonstrated the presence of rape (MTR of 10%, SD 5.4%) which was cultivated in LC-6. These reads can also be attributed to the mustard (*Sinapis alba*))—(intercultural crop) directly sown in the last crop residues four weeks before the sampling survey. These two species were not clearly distinguished. Plot 3 was one of the sites that were the most impacted by weeds. As a consequence, a large number of weed taxa were recorded in this field (i.e. bindweed, ryegrass, knotweed, healtyfoin, clover (*Trifolium*), buttercup (*Rananculus*)).

In plot 4, the last crop (wheat), accounted for 81% of MTR (SD 25%). Sugar beet, the LC-1 crop and barley, the LC-2 crop, were not identified in this field. The soil was ploughed between the LC and LC-1 and also between LC-1 and LC-2. Rape, which was never cultivated in this plot, constituted ~ 13% of MTR (SD 4%). These reads can be attributed to the intercultural crop of the same family as rape between LC-1 and LC-2. Both OTMC identified in years LC-4 (bean, *Phaseolus vulgaris*) and LC-8 (onion, *Allium cepa*) were not detected. At plot 4, eDNA analyses detected two crops and four different varieties of weeds (Bindweed, knotweed, healtyfoin and oatmeal (*Aveluna*)) (Fig. 2).

eDNA results for plot 5 indicated the dominance of the last crop, barley (MRT 46%, SD 40%) followed by the LC-1 crop (wheat)—(MRT 9.2%, SD 10.3%) and the LC-2 crop (radish)—(MRT 2.2%, SD 0.7%). OTMC identified in year LC-6 was not recorded (bean). In addition to these crops, four weeds were detected (i.e. bindweed, knotweed, healtyfoin and field gromwell (*Lithospermum arvense*)).

Plot 6 was dominated by barley (MTR—70%, SD 30%) cultivated in LC and LC-1. Wheat which was cultivated in LC-2 was not evident in the samples. The soil was ploughed between LC and LC-1 as well as between LC-1 and LC-2. The OTMC identified in LC-5 (rape) was detected in low proportions (MTR 5%, SD 2.7%). Five weed species (i.e. bindweed, knotweed, healtyfoin, clover and field gromwell) were identified along with two additional crop species (barley and wheat).

#### Conservation farming

**Figure 3:**
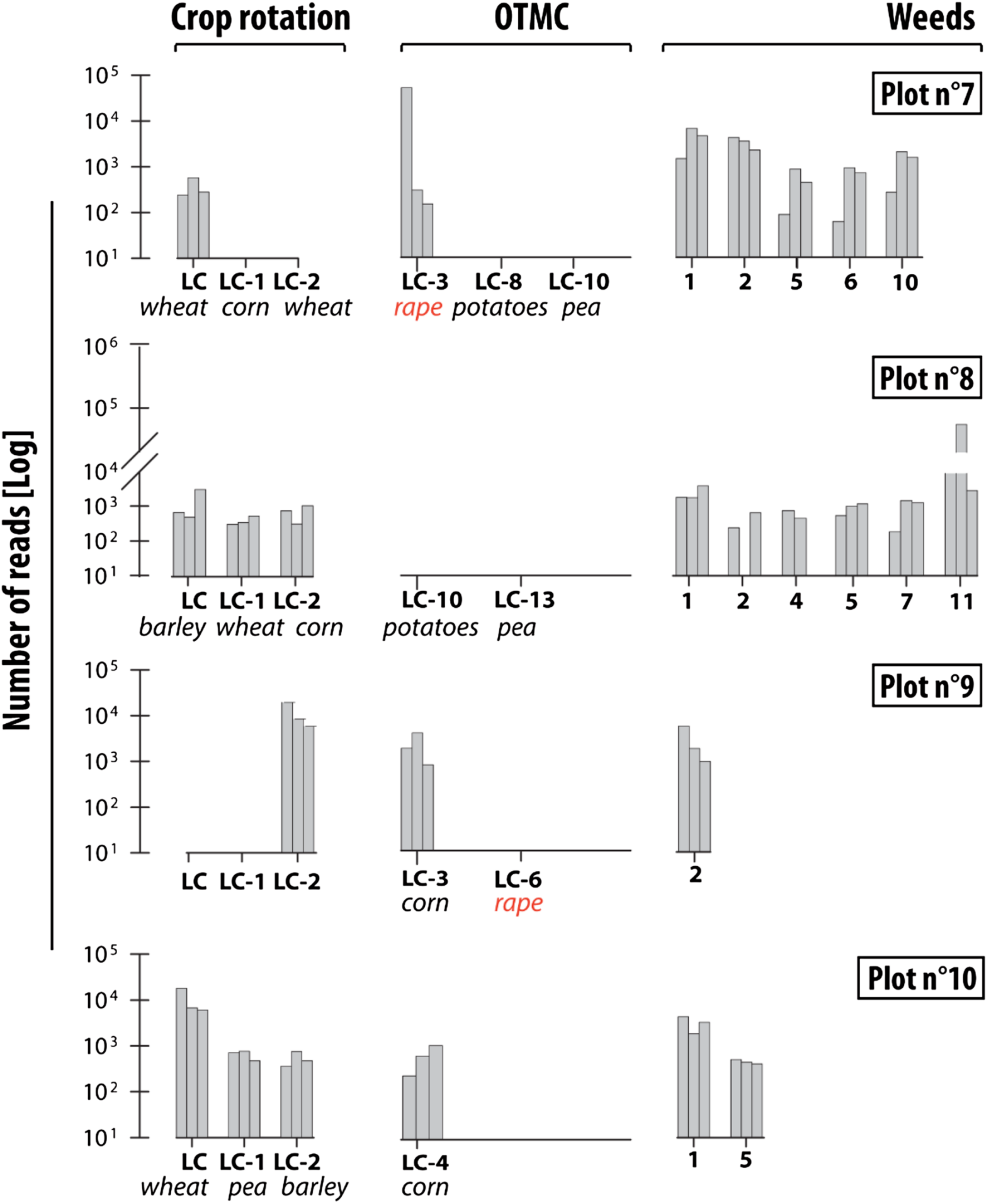
Abundance of crops and weeds taxa identified by eDNA analyses under conservation farming (i.e. no-tillage). The 3 bars correspond to the 3 replicates for each site. LC corresponds to the last crop and LC-x to the crop cultivated x year before the last crop. OTMC corresponds to Time Marker Crops (1) bindweed (*Convolvulus*) (2) ryegrass (*Lolium perenne*) (3) black nightshade (*Solanum nigrum*) (4) knotweed (*Fallopia convolvulus*) (5) healtyfoin (*Onobrychis viciifolia*) (6) clover (*Trifolium*) (7) buttercup (*Rananculus*) (8) oatmeal (*Avenula*) (9) field gromwell (*Lithospermum arvense)* (10) Artemisia (*Artemisia vulgaris*) (11) Non attributed, family of Bryaceae.

On the first field (plot 7), although wheat was grown in LC and LC-2 (wheat), this crop was only detected at low levels (MTR 1.2%, SD 0.6%). Corn cultivated in LC-1 was not identified (Fig. 3). Rape, which was cultivated in LC-3, had the highest MTR (MTR 58%, SD 99%). Barley was also detected (MTR 7%, SD 4.5%). To the best of our knowledge, barley has never been cultivated in this plot during at least 25 years, but it is cultivated in a nearby field (distance from the barley field ≈ 10 m). The most abundant weeds specie was bindweed (MTR 14%, SD 8.7%). In total, five different weeds were detected on this plot (bindweed, ryegrass, artemisia (*Artemisia vulgaris*), knotweed, healtyfoin). The OTMC planted in LC-8 (potatoes, *Solanum tuberosum*) and LC-10 (peas) were not identified by eDNA analyses (Fig. 3).

For plot 8, results indicated the dominance of a moss (non-attributed species of *Bryaceae family*: MTR 77%, SD 88%). The three last crops were identified, respectively barley (LC—MTR 5%, SD 4.7%), wheat (LC-1—MTR 2%, SD 1.4%) and corn (LC-2—MRT 2%, SD 1.2%). In addition to these crops, six weeds were recorded on this site (i.e. bindweed, healtyfoin, knotweed, clover, ryegrass and a non-attributed species from *Bryacea family*). OTMC known in LC-10 (potato) and LC-13 (pea) were not detected.

Results obtained on plot 9 indicated the detection of the wheat cultivated in LC-2 (MTR 66%, SD 35%) and one weed (ryegrass: MTR 21%, SD 12%). The last crop cultivated in LC and LC-1 (barley) was not detected. OTMC known in LC-3 (corn) was identified at this site (MTR 10%, SD 8%).

In the last plot (plot 10), the three last crops cultivated were detected, respectively wheat in LC (MTR 61%, SD 39%), peas in LC-1 (MTR 4.6%, SD 3.3%) and barley in LC-2 (MTR 3.1%, SD 3.9%). OTMC corn cultivated for the last time in LC-4 (MTR 3.9%, SD 3.3%) was detected. In addition to these crops, two weeds were detected (bindweed and healtyfoin).

#### Weed population

A large variety of weeds were identified by the eDNA analysis in addition to the main cultivated plants. Among these weeds, bindweed was identified in 100% of the fields under conventional farming and in 75% of the fields under no-tillage. This species was more abundant than the crop cultivated during LC-1 in 78% of cases. Furthermore, ryegrass was detected in 34% of the fields under conventional farming and in 75% of those under no-tillage. Ryegrass detection was particularly high in plot no. 2 where the MTR of this weed was 43% (SD 68%), which is similar to that of the last crop (MTR 40.5% SD 52%). In addition to these two ubiquitous weeds, other weed taxa were found in a large number of fields (a total of 11 different weeds were recorded in the Fromonvilliers site). Among these species, knotweed was detected in six plots and, again, healtyfoin was detected on seven plots (Figs. 2 and 3).

### eDNA as a marker of land cover

eDNA results obtained for comparing the relative abundance of the last observed crop allow the identification of the last species in 84% of fields. Under grassland, forest and channel bank environments, the expected dominant species were detected as the most abundant. Results also showed the detection of grapevine eDNA. This species is no longer cultivated at this site, but it was cultivated until 65 years ago and was identified in 45% of the sampled plots including grassland and channel bank environments.

#### Cropland

**Figure 4:**
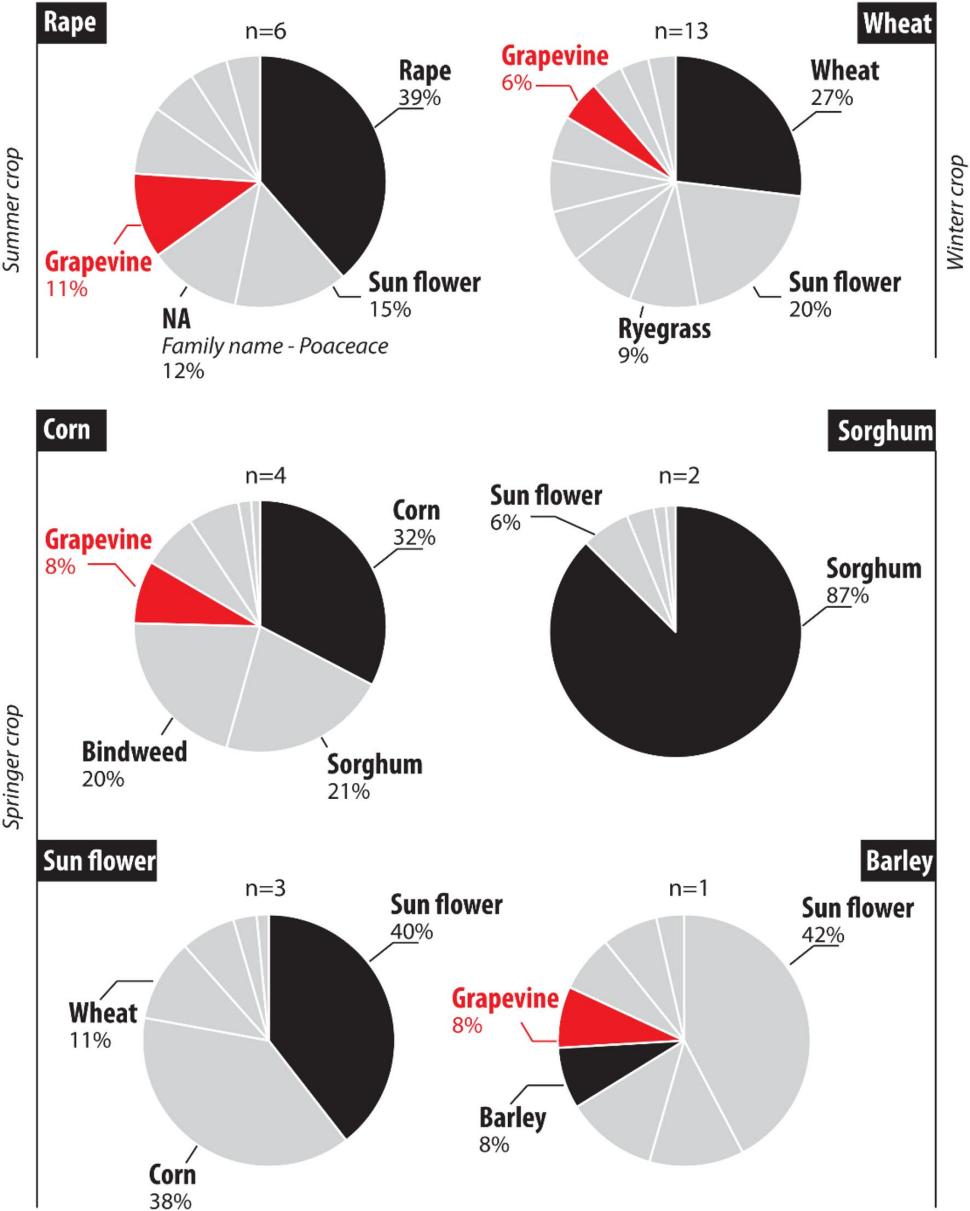
Expected versus identified species using eDNA analyses for the Louroux site. Individual pie chart corresponds to the average values per crop type (n = number of plot(s) sampled per crop type). The last cultivated crop was highlighted in black. *NA* Non attributed species.

During the fieldwork survey, rape was the only summer crop found in the Louroux catchment. eDNA results obtained for the samples collected in fields planted with rape (n = 6) showed that this species was identified in all the sampled plots. However, a high variation in abundance was observed for this species, with a rape contribution of MTR 34% (SD 31%)—(Fig. 4). Rape was detected as the most abundant species in 67% of the plots. Other taxa detected in these fields were dominated by weeds (e.g. knotweed, MTR 8%, SD 9%)—(Fig. 4).

Wheat was the main winter crop cultivated in the Louroux catchment during the survey. This species was detected in all the fields sampled (n = 12) with an abundance ranging between 1 and 96% (Fig. 4). In winter crops, eDNA analyses led to the identification of 11 different species. Among these, the four most abundant taxa detected were wheat (MTR 23%, SD 33%), sunflower (MTR 17%, SD 20%), ryegrass (MTR 8%, SD 8%) and vetch (MTR 7%, SD 8%). Ryegrass was identified in 75% of the plots, rape in 58%, grapevine in 33% and corn in 25% of the fields. Wheat was identified as the dominant taxon in only two plots (Fig. 4).

A large variety of crops were sown in the Louroux site during spring. In plots where corn was planted, this species was identified in three of the four sampled plots. Contribution of this species ranged between 0 and 57%. Sorghum was the most abundant in the plot where the corn was not identified. Among the four most abundant crops detected in the four sampled sites, corn had the highest occurrence (MTR 30%, SD 23%), followed by sorghum (MTR 20%, SD 39%), bindweed (MTR 20%, SD 23%) and grapevine (MTR 7%, SD 3%). In plots previously cultivated with sorghum, this species was the most abundant (MTR 74%, SD 9%) followed by bindweed (MTR 3%, SD 1%). In plots previously cultivated with sunflower, this species was detected in all the sampled sites (n = 3) (Fig. 4). Contribution of this plant to the MTR ranged between 3 and 60%. It was the most abundant taxon in two of the three samples (MTR 32%, SD 29%). In addition, corn (MTR 31%, SD 53%), wheat (MTR 9%, SD 12%) and ryegrass (MTR 6%, SD 5%) were also detected. Only one field previously cultivated with barley was sampled. In this plot, the barley contribution amounted to 8%. Sunflower was the most abundant taxon detected in this land cover (MTR 30%) followed by ryegrass (MTR 11%), barley and wheat (MTR 6%)—(Fig. 4).

#### Uncultivated land

**Figure 5:**
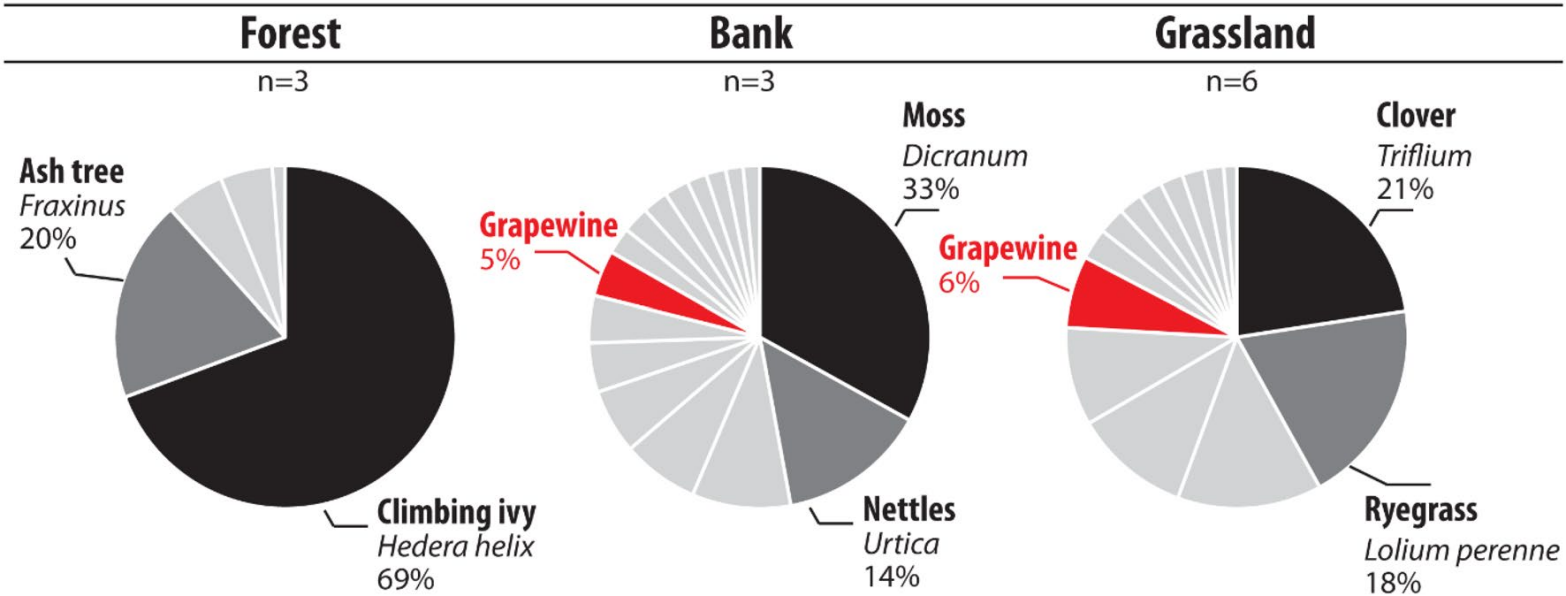
Expected versus identified taxa in forest, river channel bank and grassland environments using eDNA analyses for the Louroux site. Individual pie chart corresponds to the average values per environment type. The more abundant species were highlighted in black and dark grey.

eDNA results obtained in forested areas (n = 3 samples) showed the dominance of trees (*Fraxinus—*MTR 18%, SD 32%), shrubs (*Rhamnus—*MTR 5%, SD 7%) and climbing ivy (*Hedera helix*—MTR 66%, SD 26%) (Fig. 5). Climbing ivy was the most detected in two of the three collected samples. Trees and shrubs were the second most abundant plants. In the forest plots, six species were detected. Importantly, no cultivated plant was identified under this land use.

Seven species were identified in samples collected in permanent or temporary grassland plots (n = 6). Among them, ryegrass (MTR 18%, SD 18%), clover (MTR 21%, SD 34%) and alfalfa (MTR 13%, SD 32%) were the dominant species. These taxa were respectively detected in 84%, 33%, 15% of the plots. Grapevine was detected in 65% of the samples (MTR 6%, SD 8%), and rape was detected in 50% of the plots (MTR 10%, SD 16%).

A total of 14 different taxa were identified in channel banks (n = 3). The three samples were all characterized by the detection of specific species. The most abundant plants recorded in this environment were ryegrass (MTR 8%, SD 14%), moss (MTR 30%, SD 50%) and nettle (*Urtica—*MTR 13%, SD 22%). Importantly, no cultivated plant was detected in this land use (Fig. 5).

## Discussion

### Modern landscape detection in agricultural soils: practices effects

eDNA analyses are a powerful tool to detect recent land cover in agricultural environments. At the Fromonvilliers site, the last crop was identified in 100% of the samples in plots under conventional farming. The results obtained for these fields demonstrated the impact of soil preparation on the detection of eDNA. After tillage, the crop residues of the plant cultivated during the last year are buried in the soil (20–30 cm depth) whereas the degraded residues of the previous crop (in LC-X) could be brought back to the surface. In the current research, samples were collected in the upper 5 cm layer of the soil. This likely explains why the species corresponding to the buried plant residues was not detected. This is illustrated by the results obtained in plots 1 and 4 (Fig. 2): the crops cultivated in LC and LC-2 were recorded whereas the buried crop in LC-1 was not detected. This observation was also made for plot 6 where the LC-2 crop was not detected after tillage. For plots 2 and 3, despite ploughing, the LC-1 crops (i.e., artificial grassland and corn) were recorded in low proportions. Detection of these land covers can be explained by the large amount of crop residues that are more difficult to incorporate into the soil.

Under no-tillage (i.e. conservation farming), the last cultivated plant was detected in 75% of the sites with a lower number of reads than in conventional farming. For example, the wheat cultivated in the LC has an average MTR of 55% (SD 23%) in conventional farming whereas this contribution dropped to 31% (SD 43%) in no-tillage conservation farming (Table 1). The lower number of reads under conservation agriculture suggests a different rate of crop residue degradation depending on the farming practices (i.e. higher in conservation farming than in conventional farming).

**Table 1:** Summary of the crop identification in the 10 studied plots of the Fromonvilliers site.

		Plot 1	Plot 2	Plot 3	Plot 4	Plot 5	Plot 6	Plot 7	Plot 8	Plot 9	Plot 10
LC	MTR (%)	95.3	40.5	42.2	80.8	46.3	69.3	1.2	4.6		61.4
	SD (%)	17.6	52	10.1	24.9	35	29.8	0.6	4.7		39.1
LC-1	MTR (%)		43	1.1	OTMC—ND	9.2			2.3		4.6
	SD (%)		68	1.7		10.3			1.4		3.3
LC-2	MTR (%)	1.3	42.6			2.2			2.3	66.1	3.1
	SD (%)	0.75	31			0.7			1.2	35.9	3.9
LC-3	MTR (%)		42.6					58		10.14	
	SD (%)		31					99.2		8.14	
LC-4	MTR (%)			1.15	OTMC—ND						3.93
	SD (%)			0.68							3.3
LC-5	MTR (%)		2.5				4.9				
	SD (%)		1.7				2.7				
LC-6	MTR (%)			9.6		OTMC—ND				3.1	
	SD (%)			5.5						2.7	
LC-7	MTR (%)		1								
	SD (%)		0.75								
LC-8	MTR (%)	OTMC—ND			OTMC—ND			OTMC—ND			
	SD (%)										
LC-9	MTR (%)		OTMC—ND								
	SD (%)										
LC-10	MTR (%)	OTMC—ND						OTMC—ND	OTMC—ND		
	SD (%)										

*OTMC* One-time marker crop, *MTR* mean total reads, *SD* standard deviation, *ND* non-detected.

Crop residues incorporated into the soil by ploughing were moved to a more favorable environment for microbial activity compared to those residues remaining on or nearby the soil surface.^23^ These residues, commonly found under conventional tillage, had a larger labile component and decomposed at a faster rate compared to that prevailing under no-tillage.^24^ DNA extracted from cells by microbial activity are therefore less exposed to oxygen within the soil that when they remain exposed at the soil surface where chemical degradation induced by oxygen is more effective.

### DNA persistence

In conventional farming, eDNA analysis was able to detect plants cultivated up to 8 years before the survey (Fig. 2). The highest detection generally occurred for plants cultivated a maximum 3 years before the sampling campaign (LC-3) (Table 1). Similar observations were made for crops under conservation farming (Fig. 3). The oldest plant detected in Fromonvilliers site corresponds to the OTMC corn cultivated in LC-7. This species was also identified as the oldest OTMC in plots 9 and 10 (respectively in LC-3 and LC-4). Presence of older corn DNA can be explained by the slower rate of degradation than most of the cereals cultivated in this area. This rate of decomposition is greatly impacted by the C/N ratio, as a plant with a lower C/N ratio will degrade faster than a species with a higher C/N ratio as it will be more accessible for microorganisms.^25^ In addition, rape was also detected in a large number of plots as one of the oldest detected plant (plots 5, 6 and 7—Figs. 2 and 3). In this study, rape and mustard were not distinguished: they both belong to the *Brassicaceae* family. Mustard plant is used as a nitrogen trap to comply with the European legislation,^26^ and currently it is one of the most widely used intercrop species in France.^27^ The detection of rape in some plots could probably be attributed to more recently grown mustard. Under both farming practices, crop species cultivated during the 1970s and 1980s were neither detected.

In the Louroux catchment, where both conservation and conventional practices are applied, the last cultivated crops were detected in 96% of cases and, in 29% of these, they provided the most abundant taxon. In the remaining cases, weeds and crop rotation heads were generally found. For instance, in plots previously cultivated with wheat or barley, a large proportion of reads attributed to sunflower, corn and vetch was found. These species are often used as crop rotation heads (LC-1) in France just before planting cereal crops (LC) because of their high ability of carbon restitution.^28^ Wheat is generally sown after a superficial soil ploughing in these crop head residues. Results obtained in the current research suggest the good preservation of the last crop eDNA in association with that of the crop rotation head.

The preservation of eDNA (> 60 years) was demonstrated in the Louroux site by the detection of grapevine. This plant was identified in 46% of the cultivated plots, with an average MTR of 10% (SD 12%)—(contribution ranging between 2 and 46%). Grapevine was also identified in 67% of the grassland plots of the Louroux site and in one channel bank sample although it was not detected in the forest plots. To the best of our knowledge, the cultivation of grapevine has progressively disappeared from this catchment during the twentieth century. This land use occupied 2% of the catchment surface area by 1955 and it has completely disappeared nowadays. Unfortunately, no information is available for estimating the surface covered by this crop at the beginning of the twentieth century. Unlike the absence of detection of ancient crops at the Fromonvilliers site (< 8 years), the grapevine signal remains detected in a large number of plots across the Louroux site. Preservation of its eDNA can be explained by the high quantity of lignin material with a decomposition rate slower than that of cereals, with ~ 15% of lignin in wheat straw^29^ and classically between 15 and 40% in woody material^30^). The results suggest that residues of grapevine wood can persist for long periods, and their degradation throughout time can keep elevated the amount of eDNA in the soil. The long term persistence of residues of some species could partly explain the temporal inconsistence recorded in some palaeoenvironmental studies.^14,22^

In addition to the crop taxa detected with the eDNA analyses, results obtained at the Fromonvilliers and Louroux sites both indicated a significant contribution of weeds to the total number of reads. Weeds were characterized by a larger diversity than the cultivated species in almost all the studied plots (90% at the Fromonvilliers site and 50% for the Louroux site), and were identified with more eDNA reads in a large number of plots (e.g. in 18% of for the plots sampled in the LOU site). Among the most abundant weed species detected in the two sites, bindweed, knotweed and ryegrass were the most dominant species. In the Fromonvilliers site, the widespread occurrence of bindweed and ryegrass was found, with their identification in 90% and 40% of the plots, respectively. These weeds were also commonly observed in the Louroux site; ryegrass has been detected with eDNA analyses in 75% of the plots while bindweed was detected in 71% of the fields. In France, 52 taxa were documented to be resistant to herbicides.^31^ Among these, ryegrass is resistant to various herbicides particularly in the framework of crop rotations including wheat, the black nightshade is resistant to herbicides since 1979 (Atrazine) and a moderate resistance of oatmeal was also reported.^31,32^ To the best of our knowledge, resistances were not detected in France for bindweed and knotweed. The number of weeds detected under both farming practices are similar (with an average of four weeds under both conservation and conventional farming) with eight different species identified in conservation practices and nine in conventional practices (Figs. 2 and 3). This result demonstrates that in addition to the reconstruction of the recent land use and cover history, eDNA analyses can provide additional information on the occurrence of weeds in cultivated land.

### Perspectives and limitations

Environmental DNA analyses can be extremely useful for a wide range of research applications. This approach is particularly powerful for understanding the land use and land cover source contributions to sediment transiting river systems and accumulating in lakes and ponds through the design of improved sediment fingerprinting approaches targeting the specific crop types supplying sediment.^20^ Furthermore, as eDNA has been recorded in sediment accumulating in lakes over long periods,^22^ the current research shows that this technique can be used to reconstruct the impact of the intensification of agricultural practices and the associated land cover change during the twentieth century on soil erosion.

Nevertheless, particular attention should be paid to the potential release of past cultivated crop eDNA stored in soil, in particular hardly degradable eDNA from grapevine or other woody species.^22^

The targeting of the exact plant species grown on the soils delivering sediment to the riverine and lacustrine systems through the analysis of eDNA is particularly powerful and promising to improve our understanding of sediment transfer processes and to design effective erosion control measures. Furthermore, the current research demonstrated that this technique was able to identify weeds, which opens novel perspectives to investigate the resistance of these plants to herbicides by studying their detection in sedimentary sequences.

## Site and methods

### Study sites

Fieldwork was conducted in June and August 2017 in two cultivated areas of the southwestern part of the Parisian basin (France), in the Seine and Loire River basins (Fig. 1). These two sites, respectively the Louroux and Fromonvilliers sites were both characterized by a flat topography (slope < 1%, average elevation ~ 110 m a.s.l) and an oceanic climate with precipitation well distributed throughout the year, ranging from 620 mm at Fromonvilliers to 680 mm at the Louroux site (National meteorological center). The Fromonvilliers site was selected because records of cultivated crops and the type of farming practices implemented were available for a set of 10 agricultural fields for the last 40 years. The Louroux site was chosen because the rivers draining to the pond at the outlet are being monitored (i.e. river flow, sediment concentrations)^33,34^. Soils are very homogeneous at the Fromonvilliers site (Calcosols, with depths ranging from 1 to 4 m). Although several soil types are found in the Louroux site (Neoluvisol, Calcosol, Caclisol), they are all relatively shallow (< 1 m depth) and they have a similar sensitivity to erosion and are prone to surface crusting.

Both sites are dominated by intensive farming with conventional or conservation agriculture practices. Conventional farming mainly relies on soil ploughing (< 25 cm depth), which is usually performed before planting spring crops. In the conventional system, direct seeding is exclusively applied for drilling wheat after planting specific crops during the previous year (e.g. rape (*Brassica napus*), sugarbeet (*Beta vulgaris*), pea (*Pisum*), bean (*Phaseolus vulgaris*), sunflower (*Helianthus annuus*), or sorghum (*Sorghum bicolor*)). In other fields, conservation farming based on no-tillage and direct seeding in the previous crop residues is implemented. Four fields sampled in the Fromonvilliers site have been cultivated under no-tillage: one for 4 years (plot 7) and the three other for the last 25 years (plots 8 to 10).

Land cover change at the Fromonvilliers site was very limited during the last century, with cropland dominating the landscape. In contrast, the Louroux site has a more complex land cover history. Before WWII, this wetland area was mainly occupied by grassland and livestock farming with the occurrence of grapevines in limited parts of the catchment. For example, grapevines occupied 2% of the land use in 1955 according to the oldest agricultural census available. After 1950, major changes occurred to drain these hydromorphic soils through the design of tile drains, streams and ditches in order to convert grassland and wetlands into cropland^35,36^.

### Soil sampling strategy

At the Fromonvilliers site, soil surface samples were collected at ten plots with a well-documented history of crop rotation for the last 40 years. The plantation of some atypical crops occurred during well-defined periods and they were no longer cultivated afterwards (e.g. hemp production (*Cannabis sativa*) during the 1970s or alfalfa (*Medicago sativa*) during the 1980s). Crops that were planted only once per rotation (e.g. pumpkin (*Cucurbita maxima)*, bean (*Phaseolus vulgaris)*, radish (*Raphanus raphanistrum),* potatoes *(Solanum tuberosum*), onion *(Allium cepa)*) were identified as potential one-time marker crops (OTMC). The detection of eDNA attributed to a OTMC or to a crop that is no longer cultivated was used to quantify the soil memory effect as their periods of cultivation are known. At each sampling point, five subsamples (~ 5 g per subsample, sample depth < 5 cm) were retrieved using a trowel sterilized with a blowtorch following the recommendations provided by Taberlet et al*.* (2012). These samples were aggregated and homogenized. Sampled material was stored in a sealed plastic container with silica gel bags for in-situ drying. In each plot, triplicate samples were taken resulting in a total of 30 samples collected at the Fromonvilliers site.

For the Louroux site, 41 samples were taken in selected fields across the catchment in order to cover the large variety of land cover and crop species currently found in this catchment including cropland (samples were collected in plots previously occupied by summer crop (rape, n = 6 samples), winter crop (wheat n = 13 samples) and springer crops (corn n = 4, sunflower n = 3, sorghum n = 2 and barley n = 1 sample)) and grassland (n = 6). In addition, samples were collected in forests (n = 3) and along river channel banks (n = 3). One sample was collected in each of the selected sites using the aforementioned procedure outlined for sampling in Fromonvilliers. Although the detailed crop rotations are not known, agricultural census data are available for this catchment since 1955. For both sites, the chronology of the crops cultivated were ranked from the last crop (LC), corresponding to the year of the LC present in the plot at the sampling date, to LC*-*X, the crop cultivated X years before the sampling survey. For example, LC-3 is a crop that was cultivated 3 years prior to the last crop (LC) cultivated before the sampling date.

### Extracellular DNA analyses

Soil eDNA targeting extracellular eDNA was extracted following the procedure described in Pansu et al.^38^ and Evrard et al.^20^ For each sample, approximatively 15 g of air-dried soil was mixed with 15 ml of saturated phosphate buffer (Na_2_HPO_4_; 0.12 M, pH ≈ 8) for 15 min. Two ml of the mixture was centrifuged (10 min at 10,000 g) and ~ 400 μl of resulting supernatant was kept as starting material for extraction using the NucleoSpin Soil kit (MACHEREY–NAGEL, Düren, Germany), skipping the cell lysis step and following manufacturer's instructions.^37^ The extracted DNA was eluted in 100 μl of SE buffer and used as PCR (polymerase chain reaction) template. Seven extraction controls were also conducted, in which the complete extraction procedure was applied to the NucleoSpin columns, without adding the supernatant. The eDNA of vascular plants was amplified with the g-h primers, which amplify a short region of chloroplast DNA, which is variable enough to discriminate between plant taxa at the family, genus or species level in the best case.^39^ These primers are highly conserved for Spermatophyta (seed plants^39^). In-silico analyses showed that they are expected to amplify ~ 99% of plant species,^4^ and several in-vitro analyses confirmed that they provide robust measurements of plant diversity e.g.^8,38,40^ Additionally, six PCR controls containing PCR mix and no DNA template, along with six positive PCR controls were performed.^13^ Each soil sample and control was amplified in four PCR replicates.^41^ The sequencing was conducted by 2 × 125—base pair pair-end sequencing on an Illumina HiSeq 2,500 platform. The sequences of DNA were filtered with OBITools software^42^ following the procedure detailed in Pansu et al*.*^38^ Plant sequences were assigned using the reference database of the vascular plants found in France.

For each taxon, the sum of the reads of the four replicates by sample was calculated. The proportion of reads assigned to a given taxon is often positively correlated to the relative abundance of this species.^12^ Nevertheless, as detailed in Ficelota et al.^43^ this relationship can be biased by multiple factors and results need to be interpreted with caution.

To limit the risk of false positives, taxa with a total number of reads lower than 1,000 were removed from further analysis. Additionally, if a taxon was not detected at least in two of the three soil samples collected in each plot, then it was not taken into account.^13^ For the data interpretation, the mean total reads (MTR) expressed in a percentage (%) and the standard deviation (SD %) were calculated for each species using the 12 DNA sequences available in every plot of the Fromonvilliers site (three samples by plot and 4 repetitions by samples) and using the four DNA sequences for the Louroux site.
